# Psychometric properties of the Chinese version of Attitudes and Beliefs about Cardiovascular Disease Risk Perception Questionnaire

**DOI:** 10.1038/s41598-022-24620-9

**Published:** 2022-11-24

**Authors:** Zhiting Guo, Chuanqi Ding, Wen Gao, Junyi Hong, Jiaying Tang, Yuping Zhang, Jingfen Jin

**Affiliations:** 1 The Second Affiliated Hospital of Zhejiang University School of Medicine (SAHZU), Nursing Department, No. 88 Jiefang Road, Shangcheng District, Hangzhou, 310009 Zhejiang Province China; 2 Changxing County People’s Hospital, Emergency Department, No. 66 Taihu Middle Road, Changxing County, Huzhou, 313199 Zhejiang Province China; 3 Stomatology Hospital, Zhejiang University School of Medicine (ZJUSS), Nursing Department, No. 166 Qiutao North Road, Shangcheng District, Hangzhou, 310020 Zhejiang Province China; 4 Key Laboratory of the Diagnosis and Treatment of Severe Trauma and Burn of Zhejiang Province, Hangzhou, 310009 Zhejiang Province China

**Keywords:** Cardiology, Health care, Risk factors

## Abstract

Cardiovascular disease (CVD) is the leading cause of global mortality and disease burden. The perceived risk of CVD, a central psychological construct, may affect health behavior change and maintenance, such as lifestyle modification and medication adherence. Risk perception varies based on the knowledge of risk in the context of cultural health-world views. Little is known about CVD-related knowledge and risk perception in China. The aim of this study is to cross-culturally translate, adapt, and evaluate the psychometric properties of the Attitudes and Beliefs about Cardiovascular Disease (ABCD) Risk Perception Questionnaire in Chinese. The translation and cross-cultural adaptation process followed established guidelines. A cross-sectional study of 318 adults between April and May 2022 was conducted in Zhejiang province. The study evaluated the item- and scale-level psychometric properties and validity indices of the ABCD risk perception questionnaire. The exploratory and confirmatory factor analyses of the risk scale supported a three-factor solution that accounts for 69.63% of the total variance, corresponding to risk perception (F1), perceived benefits and intention to change physical activity (F2), and perceived benefits and intention to change dietary habits (F3). Adequate content validity (I-CVI = 0.852–1.00, S-CVI = 946) was ensured by expert panel. The internal consistency of the dimensions showed good results ranging from 0.801 to 0.940 for Cronbach's *α*, and 0.853 to 0.952 for McDonald’s ω. The item analysis of knowledge dimension indicated that the item difficulty index was 0.440 to 0.852, the item discrimination index was 0.572 to 0.707. This study confirmed that the Chinese version of the ABCD risk perception questionnaire has good psychometric properties in terms of measuring CVD-related knowledge and risk perception in the Chinese adult population, which can lead to the development of individually tailored CVD-risk reduction intervention programs or risk communication programs by health providers.

## Introduction

### Cardiovascular disease prevention in China

Cardiovascular disease (CVD) is the leading cause of global mortality and a major contributor to disability^1^ as well as the leading cause of death and disease burden in China, accounting for 40% of deaths^2^. An unhealthy lifestyle, including physical inactivity, poor dietary habits, tobacco use, and excessive alcohol consumption, has been identified as an alterable risk factor for CVD^3^. As such, CVD risk can be reduced through the adoption of a healthy lifestyle, which has been recommended by CVD primary and secondary prevention guidelines^4,5^. Nevertheless, a large number of individuals have a low awareness of CVD risk factors and poor treatment and control rates of these major risk factors^2^.

### Relationship between CVD knowledge, risk perception, and healthy lifestyle

Understanding of individual risk and the potential benefits of prevention is a prerequisite for the implementation of preventive measures^6^. Further, active involvement of the target population is critical for CVD prevention interventions. The perceived risk of CVD, a central psychological construct, may affect health behavior change and maintenance^7,8^; specifically, individuals who perceive themselves as having a higher risk of CVD are more likely to adopt a healthy lifestyle or adhere to prevention measures for CVD^9,10^. Although the perception of risk can be formed by comparing risk-factor knowledge with one’s lifestyle and experience^11^, risk perception varies based on knowledge of the risk in the context of cultural health-world views^12^. Without taking these views into consideration, people may under- or overperceive their CVD risk. If they over-perceive their CVD risk, then people may be thrust into situations that they are ill-prepared to handle and suffer psychological strain; in turn, if they under-perceive their CVD risk, individuals may ignore the risk and be less likely to modify their health behaviors^13^.

### CVD knowledge and risk perception measurements

Poor knowledge or misperception of CVD risk impedes the attainment of better health outcomes. Thus, the measurement of CVD knowledge and risk perception is essential to promoting a healthy lifestyle intervention. A single-item measurement of absolute or relative CVD risk has been used in many studies in the past four decades^14–16^, which is not sufficient to assess the multiple dimensions of risk perception^17^. The Perception of Risk of Heart Disease Scale (PRHDS)^18^ or the Coronary Risk Individual Perception Scale (CRIP)^8^, as a type of specific risk perception measurement, focuses mainly on heart disease and does not measure CVD risk knowledge; as such, it is not appropriate for risk perception evaluation. In response to this need, Woringer et al. constructed the Attitudes and Beliefs about Cardiovascular Disease (ABCD) Risk Perception Questionnaire to measure CVD knowledge and risk perception among individuals in England^19^. The ABCD Risk Perception Questionnaire consists of 26 items classified into four subscales: Knowledge of CVD Risk and Prevention, Perceived Risk of Heart Attack/Stroke, Perceived Benefits and Intention to Change Behavior, and Healthy Eating Intentions. The ABCD Risk Perception Questionnaire has been translated to other languages and applied in Hungary^20^, Belgium^21^ and Malay^22^; and research has shown that this scale had good psychometric properties in terms of accessing CVD-related knowledge and risk perception. Further, the Packages of Interventions for Cardiovascular Diseases in Europe and Sub-Saharan Africa identified the ABCD Risk Perception Questionnaire as a potential tool to measure CVD knowledge and risk perception prior to and after an intervention^21^.

### Purpose of the study

To the best of our knowledge, there is not an appropriate CVD knowledge and risk perception measurement in the Chinese cultural context. Therefore, this study aims to investigate the validity and reliability of a Chinese version ABCD Risk Perception Questionnaire. This questionnaire is a multidimensional measurement that can lead to the development of individually tailored CVD risk reduction interventions or risk communication programs by health providers.

## Materials and methods

### Study setting and participants

A cross-sectional survey was conducted and reported, following the Strengthening the Reporting of Observational Studies in Epidemiology statement^23^. A convenience sample was recruited for the study from health check center and endocrinology department of the second affiliated hospital of Zhejiang University school of medicine in Hangzhou, Zhejiang province, China. A paper-based survey and online survey platform powered by WJX (www.wjx.com) between April and May 2022 were performed. The inclusion criteria were being a Zhejiang citizen, age greater than 18 years, not undergoing treatment for a psychiatric disorder, and no barriers to communication. Before the assessment process, the participants were informed about the topic of the study and gave informed consent. The sample size was based on the recommended subject-to-item of (5–10):1, for which 130–260 participants were required. Taking into consideration a missing data rate of 10% and adequate sample size for confirmatory factor analysis (CFA)^24^, 318 participants were included.

### Measures

#### Chinese version of the ABCD Risk Perception Questionnaire

Permission was obtained from the author to translate the English version to Chinese. We conducted this procedure following the Cross-cultural Adaptation of Self-report Measures guidelines^25,26^ and consensus-based standards for the selection of health measurement instruments^27^.

##### Translation procedure

First, two native Mandarin Chinese-speaking translators who were fluent in English translated the English ABCD Risk Perception Questionnaire into Mandarin Chinese independently. Then, the two Chinese versions were compared with the original scale by a third translator. The research team discussed any discrepancies and formed a consensual Mandarin version. This consensual Mandarin version was given to two bilingual translators who were blind to the original English version and who translated the Mandarin version back into English. The back-translated versions of ABCD Risk Perception Questionnaire were compared with the original one, and the translators clarified a few ambiguities and inconsistencies.

##### Cross-cultural adaptation process

An expert panel, including a cardiologist, two nurse specialists from a cardiology department, a health management specialist, a methodologist, and a language professional, reviewed all translation reports with translators in terms of semantic equivalence, idiomatic equivalence, experiential equivalence, and conceptual equivalence until consensus was achieved. The pre-final version proposed by these experts was sufficiently close to the original one. Finally, all experts rated the relevance of each item of the ABCD Risk Perception Questionnaire to the Chinese context on a 4-point Likert scale (1 = very irrelevant; 4 = very relevant). Further, comprehensiveness and intelligibility, according to the COSMIN rating system, was determined by experts, using a 3-point scale (1 = not clear, 2 = not sufficient, 3 = sufficient)^27^.

##### Pilot testing of the pre-final version

The pre-final ABCD Risk Perception Questionnaire was tested among 40 Chinese adults according to the aforementioned cross-cultural adaptation guidance^25^. Participants took about 5 min to complete the questionnaire. In addition, each participant was invited to comment on his or her understanding of the wording, particularly in regard to confusing or unreadable statements. After minor revisions, the final ABCD Risk Perception Questionnaire was generated for psychometric evaluation.

##### Final Chinese version of the ABCD Risk Perception Questionnaire (ABCD-C)

This questionnaire consists four subscales. The Knowledge of CVD Risk and Prevention diemsion consists of eight statements about CVD risk, regarding whether the respondent agrees or disagrees with the statements with three options (True/False/I don’t know). For each item, the correct answer was scored as 1, and an incorrect or “I don’t know” answer was scored as 0. Values are summed to create a summary score that can range from 0 to 8, for which higher values indicate higher CVD-related knowledge. The other dimensions include Perceived Risk of Heart Attack/Stroke (8 items), Perceived Benefits and Intention to Change Behavior (7 items), and Healthy Eating Intentions (3 items). Answer options are presented on a 4-point scale and range from 1 = strongly disagree to 4 = strongly agree. Items 15, 21 and 26 were reverse-coded.

#### Other measures

Demographic characteristics included age, sex, marital status, education level, religion, employment status, smoking and drinking status, family history, and related variables. Subjective health status was estimated through the question, “In general, how would you rate your health status?” (1 = very poor, 2 = poor, 3 = fair, 4 = good, 5 = excellent). Smoking and drinking status was determined by the answer to the question, “What is your current smoking/drinking status?” (1 = never smoked/drank, 2 = ever smoked/drank, 3 = currently smoke/drink). In addition, one item estimated absolute CVD risk: “What do you think the risk of your getting any kind of cardiovascular disease within the next 10 years is?” by selecting a number from 1 to 10, with 0 = no risk and 10 = very high risk, a question routinely used in CVD risk perception related studies^16,28^.

### Data collection procedures

Data were collected on-site and online. Well-trained and eligible research assistants who were all nursing Ph.D. candidates collected data through face-to-face interviews. All participants were informed of the purpose of the research and that their participation was voluntary and confidential. Questionnaires with more than three blank items in the paper survey were excluded. No items were missing for the online survey because all items were required, but a completion time of less than 2 min or similar option choices invalidated the questionnaire, which was excluded. In addition, the ABCD Risk Perception Questionnaire was completed twice by twenty participants who were randomly selected from the total sample in two-week intervals to calculate the test–retest reliability.

### Psychometric assessments and statistical analysis

Scale reliability of the ABCD-C was tested by internal consistency and test–retest reliability. The degree of internal consistency is described as Cronbach’s *α* and McDonald’s ω. An item‐total corrected correlation coefficient was employed to calculate items discrimination. Test–retest reliability was assessed by an intraclass correlation coefficient (ICC), rooted in a two-way analysis of the variance in a random effect model. The 95% confidence intervals (CIs) of ICC value also reported. Both Cronbach’s *α*, McDonald’s ω and ICC values higher than 0.70 are recommended which indicate that the scale has acceptable reliability^29,30^.

Construct validity and content validity were evaluated to verify the validity of ABCD-C. The content validity of the scale was assessed by the content validity index of items (I-CVI) and the scale content validity index-average (S-CVI/Ave) based on the ratings of experts on this questionnaire. A S-CVI/Ave value of 0.9 is considered an excellent criterion and a value of 0.8 as the lower content validity limit for acceptance of the whole scale^31^, I-CVI ≥ 0.78 is considered to be appropriate if the number of experts is ≥ 6^32^. Exploratory (EFA) and confirmatory factor analysis (CFA) with maximum likelihood were performed to explore the dimensionality of items in more detail. The standardized factor loadings and an estimated of the variance in the measured variable explained by the latent variable (R^2^), together with fit statistics (χ^2^, CFI—comparative fit index, IFI—incremental fit index; TLI—Tucker-Lewis index, RMSEA—root mean square error of approximation). A CFA > 0.95, TLI > 0.90, RMSEA < 0.08 were considered acceptable^33^. Average variance extracted (AVE) was applied to assess the internal convergent validity of each factor, with a score ≥ 0.5 indicating satisfactory convergent validity^34^. The square root of AVE value exceeding each of its correlations with other factors indicate appropriate discriminant validity^34^. To evaluate the concurrent validity, the Spearman rank correlations were analyzed between the ABCD-C and single-item CVD subjective risk perception. The correlation of |*r*|= 0.10–0.30, |*r*|= 0.31–0.60, and |*r*|= 0.61–1.00 were considered low, moderate and high, respectively^35^.

Item difficulty for knowledge subscale was determined by descriptive statistics, consistent with previous knowledge studies^36^. The difficulty level index was calculated as the number of correct answers divided by the total number of answers, a higher index indicates a lower level of difficulty^37^. The difficulty level of a test is expected to be around 0.50^37^. The corrected correlations between the performance among individual items and the overall test were calculated to determine the ability of each item to discriminate high- and low-scoring participants. A correlation value of 0.5 to 0.7 indicated good discrimination power^38^.

The Statistical Package for the Social Sciences, version 26.0 (SPSS, Chicago, IL, USA), was used for statistical analysis. The data are expressed as the mean (*M*) and standard deviation (SD). Mean difference of ABCD-C scores across the sociodemographic variables were determined using one-way analysis of variance (ANOVA) and Tukey`s multiple comparison test. The effect size partial eta squared (η^2^) was calculated through the sum of squares of the effect divided by the total sum of squares; η^2^ = 0.01 indicates small effect, η^2^ = 0.06 with medium and η^2^ = 0.014 with large effect^36^. CFA was conducted using AMOS 22.0 to assess the structural validity of the scale. SPSSAU was used to conduct reliability analysis. A *p*-value less than or equal to 0.05 was considered statistically significant.

### Ethics approval

The study protocol had been approved by the Institutional Review Board of the second affiliated hospital of Zhejiang University School of Medicine (No. ID: 2022-0280).

### Informed consent

Informed consent was obtained from all subjects involved in the study. The study was in accordance with the principles of the Declaration of Helsinki.

## Results

### Translation and cultural adaptation

Slight differences were identified in sentence statements between the original and translated versions, and minor revisions were made. In Items 14, 17, and 18, “five portions of fruit and vegetables” was not easily understood by participants without a medical background; therefore, we used “500 g of fruit and vegetables,” according to the Cardiovascular Primary Prevention guidelines in China^39^. Similarly, “2–1/2 h” is not idiomatic in Chinese, so 2–1/2 h was changed to 2.5 h. In the pilot testing, 40 participants were randomly selected to identify whether the questionnaire could be understood correctly. All participants were satisfied with the number and comprehensibility of the items.

### Respondent characteristics

A total of 341 participants were approached for the study. Of these, 93 (27.3%) completed the paper-based questionnaire, and seven answers (2.0%) had more than three blanks and were excluded. A total of 248 participants (72.7%) finished the online survey, for which five responses had a completion time of less than 2 min, and 11 responses had very similar option choices for most items; thus, the data from 16 questionnaires (4.7%) were excluded. No difference in response rate was found between paper-based and online participants, other comparisons across characteristic variables were showed in supplementary file Table S1. As such, the data from 318 (93.3%) surveys were included in the final analysis. Ages ranged between 20 and 87 years (*M* = 42.04, SD = 16.89). The score of single item subjective risk perception ranged from 0 to 10 (*M* = 2.55, SD = 1.625). Table 1 displays the sociodemographic information of the participants.

**Table 1:** Characteristics of the participants.

Characteristic	*n* (%)	ABCD-C (*M* ± *SD*)			
		Risk perception (Range 0–32)	PA (Range 0–24)	Dietary (Range 0–16)	Knowledge (Range 0–8)
**Total sample**	318	17.30 ± 5.17	18.11 ± 4.16	12.76 ± 2.21	5.82 ± 2.19
**Sex**					
Male	134 (42.1)	17.64 ± 5.10	18.19 ± 3.68	12.53 ± 2.16	5.43 ± 2.22
Female	184 (57.9)	17.05 ± 5.22	18.05 ± 4.49	12.93 ± 2.24	6.11 ± 2.13
ANOVA F (*p* value)		1.000 (0.318)	0.087 (0.768)	2.533 (0.112)	7.487 (0.007)
Partial η^2^		0.003	< 0.001	0.008	0.023
**Education level**					
Junior school or below	44 (13.8)	18.57 ± 3.53	19.32 ± 3.10^a^	13.05 ± 1.84	3.39 ± 1.48^abc^
High school/specialized secondary school	59 (18.6)	18.17 ± 2.90	18.95 ± 3.11	12.69 ± 2.00	4.73 ± 2.10^ade^
Specialty/bachelor	193 (60.7)	16.80 ± 5.91	17.58 ± 4.54^a^	12.66 ± 2.36	6.52 ± 1.86^bdf^
Postgraduate or above	22 (6.9)	16.86 ± 5.15	18.18 ± 4.32	13.27 ± 2.07	7.55 ± 1.01^cef^
ANOVA F (*p* value)		2.118 (0.098)	3.159 (0.025)	0.788 (0.501)	49.367 (< 0.001)
Partial η^2^		0.020	0.029	0.007	0.320
**Ethnic group**					
Han Chinese	309 (97.2)	17.31 ± 5.17	18.07 ± 4.20	12.75 ± 2.24	5.83 ± 2.20
Minorities	9 (2.8)	17.11 ± 5.39	19.44 ± 2.29	13.00 ± 1.00	5.67 ± 2.12
ANOVA F (*p* value)		0.013 (0.911)	0.946 (0.331)	0.107 (0.743)	0.047 (0.828)
Partial η^2^		< 0.001	0.003	< 0.001	< 0.001
**Employment status**					
Employed	193 (60.7)	16.91 ± 5.73	17.89 ± 4.39	12.85 ± 2.18	6.48 ± 1.92
Unemployed	125 (39.3)	17.90 ± 4.10	18.46 ± 3.76	12.62 ± 2.26	4.81 ± 2.21
ANOVA F (*p* value)		2.807 (0.095)	1.463 (0.227)	0.882 (0.348)	50.979 (< 0.001)
Partial η^2^		0.009	0.005	0.003	0.139
**Smoking status**					
Current smoking	43 (13.5)	18.56 ± 2.76	18.19 ± 2.61	12.47 ± 1.94	4.81 ± 2.18
Non-smoking/quit smoking	275 (86.5)	17.11 ± 5.43	18.10 ± 4.36	12.81 ± 2.25	5.98 ± 2.16
ANOVA F (*p* value)		2.951 (0.087)	0.015 (0.902)	0.887 (0.347)	10.833 (0.001)
Partial η^2^		0.009	< 0.001	0.003	0.033
**Drinking status**					
Current drinking	55 (17.3)	18.58 ± 3.78*	18.49 ± 4.10	13.02 ± 2.13	5.33 ± 2.31
Non-drinking/quit drinking	263 (82.7)	17.03 ± 5.38*	18.03 ± 4.18	12.71 ± 2.23	5.93 ± 2.16
ANOVA F (*p* value)		4.112 (0.054)	0.546 (0.460)	0.896 (0.345)	3.424 (0.065)
Partial η^2^		0.013	0.002	0.003	0.011
**CVD family history**					
Yes	28 (8.8)	19.04 ± 5.78	18.79 ± 2.79*	12.89 ± 2.23	5.79 ± 2.40
No	290 (91.2)	17.13 ± 5.08	18.05 ± 4.27*	12.75 ± 2.21	5.83 ± 2.18
ANOVA F (*p* value)		3.477 (0.063)	0.800 (0.372)	0.108 (0.742)	0.009 (0.923)
Partial η^2^		0.011	0.003	< 0.001	< 0.001
**Subjective health status**					
Excellent	120 (37.7)	16.30 ± 5.74^a^	17.43 ± 5.32^a^	12.83 ± 2.62	6.53 ± 2.07^abc^
Good	154 (48.4)	17.53 ± 4.72	18.92 ± 2.99^a^	12.77 ± 1.97	5.49 ± 2.16^a^
Fair	39 (12.3)	19.49 ± 4.45^a^	17.31 ± 3.53	12.56 ± 1.68	5.26 ± 2.18^b^
Poor	5 (1.6)	17.40 ± 4.33	15.80 ± 4.08	12.40 ± 2.70	3.60 ± 1.14^c^
ANOVA F (*p* value)		4.031 (0.008)	4.121 (0.007)	0.188 (0.904)	8.519 (< 0.001)
Partial η^2^		0.037	0.038	0.002	0.075

ABCD-C, Chinese version of Attitudes and Beliefs about Cardiovascular Disease (ABCD) Risk Perception Questionnaire; RP, risk perception; PA, perceived benefits and intention to change physical activity; DH, perceived benefits and intention to change dietary habits; CVD, cardiovascular disease.

*The significance level of the mean difference is 0.05; ** The significance level of the mean difference is 0.001. Same lower-case alphabets denote a significant difference between each level of variables in Colum,* p* < 0.05.

### Psychometric analysis

#### Validity

The content validity of ABCD-C was assessed through expert consultation, according to the COSMIN Checklist^27^. The CVI for “relevance,” “comprehensiveness,” and “intelligibility” ranged from 85 to 100%. The results indicated that the content validity of the ABCD-C was excellent, with the I-CVI as between 0.852 and 1.00 and S-CVI as 0.946.

**Figure 1:**
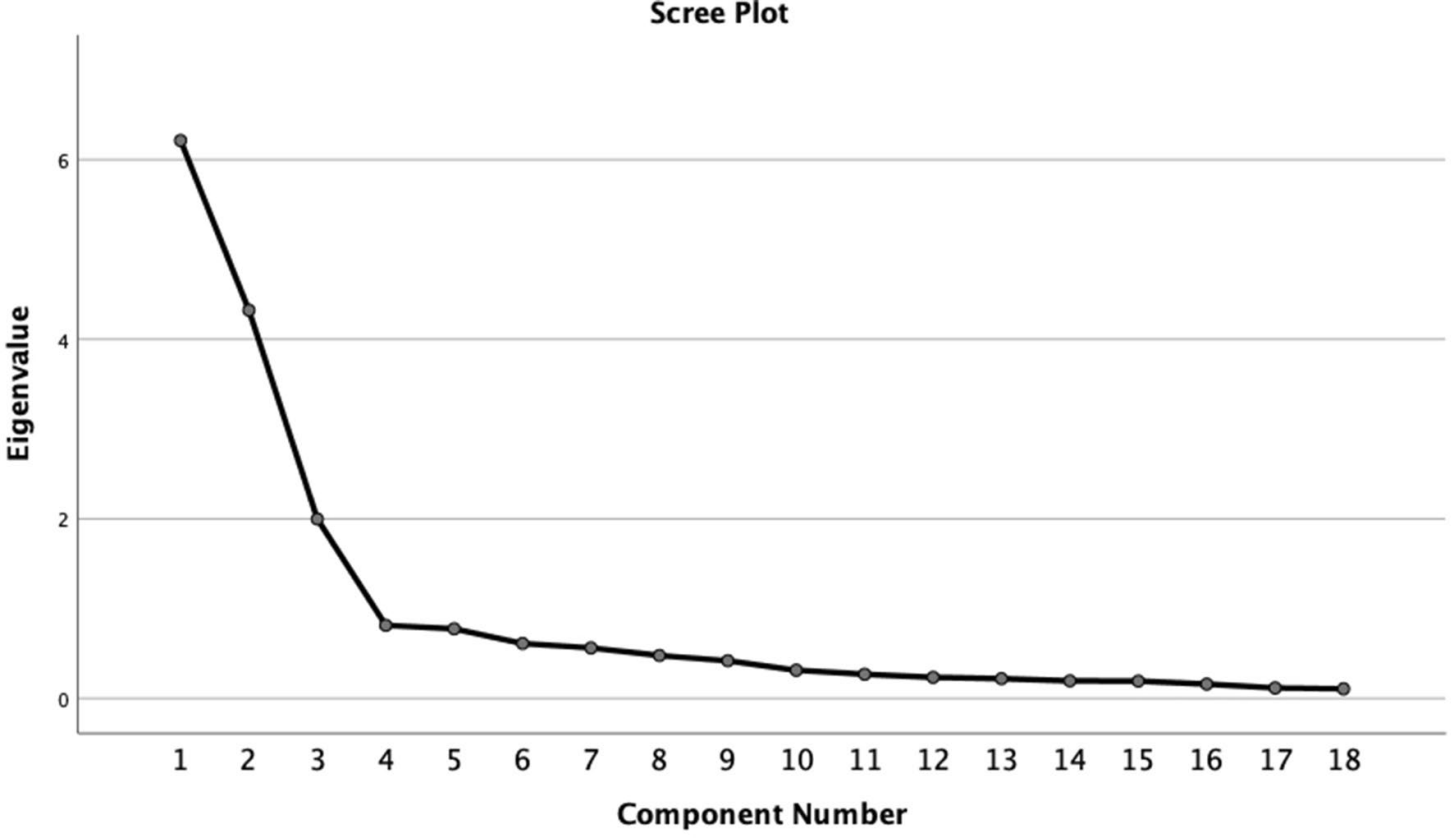
Scree plot of the exploratory factor analysis.

Structural validity was investigated via EFA. The Kaiser–Meyer–Olkin (KMO) value was 0.861, and Bartlett`s test of sphericity was significant (*p* < 0.001), which indicated that the 18-item ABCD risk perception questionnaire was adequate in terms of EFA (the Knowledge of CVD Risk and Prevention subscale had 8 items, and the data type did not qualify for factor analysis). Based on the scree plot (Fig. 1) and parallel analysis, a three-factor solution emerged with a cumulative variance contribution rate of 69.632%, although the pattern of factor loadings in our analysis was slightly different from the original one. The domains in the original ABCD Risk Perception Questionnaire were Perceived Risk of Heart Attack/Stroke (Items 1–8), Perceived Benefits and Intention to Change Behavior (Items 9–15), and Healthy Eating Intentions (Items 16–18). In our analysis, Items 14 and 16–18 had the same factor loading (Factor 3); in addition, all of these items expressed *healthy diet* semantically. Thus, we renamed the domains according to loading patterns and Hassen HY et al.’s results^21^: Factor 1: risk perception (RP), Factor 2: perceived benefits and intention to change physical activity (PA); and Factor 3: perceived benefits and intention to change dietary habits (DH) (Table 2).

**Table 2:** Factor loadings of the EFA.

Item No	Factor 1	Factor 2	Factor 3	Item score		
				Mean (SD)	Skewness (SE)	Kurtosis (SE)
09	**0.826**	0.188	0.118	2.45 (0.82)	− 0.86 (0.14)	0.89 (0.27)
10	**0.882**	0.119	0.040	2.25 (0.77)	− 0.45 (0.14)	0.77 (0.27)
11	**0.825**	0.104	0.111	2.21 (0.79)	− 0.35 (0.14)	0.73 (0.27)
12	**0.836**	0.016	0.046	2.01 (0.69)	− 0.35 (0.14)	1.04 (0.27)
13	**0.825**	0.119	0.037	2.03 (0.69)	− 0.27 (0.14)	1.17 (0.27)
14	**0.891**	0.053	0.038	2.11 (0.74)	− 0.51 (0.14)	0.35 (0.27)
15	**0.776**	− 0.083	− 0.057	2.12 (0.89)	0.31 (0.14)	− 0.08 (0.27)
16	**0.863**	− 0.070	− 0.047	2.13 (0.71)	− 0.29 (0.14)	0.49 (0.27)
17	0.132	**0.856**	0.101	2.93 (0.86)	− 1.22 (0.14)	2.27 (0.27)
18	0.064	**0.860**	0.122	2.95 (0.96)	− 1.05 (0.14)	1.72 (0.27)
19	0.008	**0.783**	0.201	3.19 (0.88)	− 1.56 (0.14)	3.17 (0.27)
20	0.072	**0.787**	0.192	2.85 (0.88)	− 0.88 (0.14)	1.14 (0.27)
21	0.040	**0.803**	− 0.023	2.93 (0.79)	− 0.85 (0.14)	1.48 (0.27)
23	0.013	**0.721**	0.252	3.26 (0.80)	− 1.33 (0.14)	2.44 (0.27)
22	− 0.006	0.190	**0.767**	3.25 (0.59)	− 0.87 (0.14)	3.70 (0.27)
24	0.195	0.188	**0.809**	3.11 (0.75)	− 0.89 (0.14)	1.79 (0.27)
25	0.041	0.189	**0.881**	3.13 (0.68)	− 0.82 (0.14)	1.86 (0.27)
26	− 0.031	0.078	**0.716**	3.27 (0.67)	− 0.76 (0.14)	0.96 (0.27)
Factor score [Median, *M*(*P*_*25*_, *P*_*75*_)]	− 0.714 (− 0.329, 0.746)	0.101 (− 0.309, 0.566)	0.005 (− 0.453, 0.570)			
Eigenvalues	5.730	4.069	2.735			
Variance explained (%)	31.833	22.603	15.195			
Cumulative variance explained	31.833	54.437	69.632			

Varimax was the applied rotation method. Factor loadings with absolute values higher than 0.5 are in bold. Factor 1: Risk perception, Factor 2: Perceived benefits and intention to change physical activity, and Factor 3: Perceived benefits and intention to change dietary habits.

**Figure 2:**
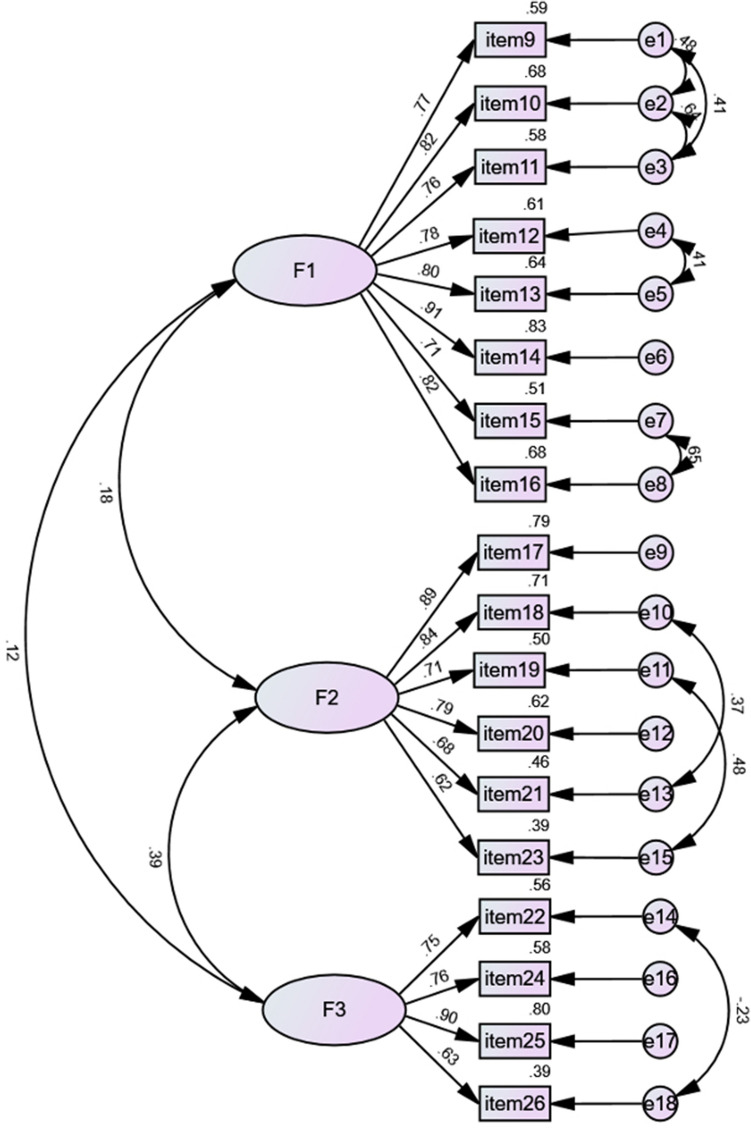
Factor structure model and estimated value of the standardized loadings of ABCD-C.

We further performed CFA to confirm the EFA-derived 3-factor structure. The initial model indices suggested an inadequate model fit, and eight paths of covariance between errors were added based on the modification indices. The adjusted model had satisfactory fit indices: χ^2^ = 348.69, *df* = 124, χ^2^/*df* = 2.812, *p* < 0.001; CFI = 0.948, IFI = 0.948, TLI = 0.936; RMSEA = 0.076 (90% CI 0.066 to 0.085). The CFA path diagram of the ABCD-C plotted with standardized parameter estimates is displayed in Fig. 2. We also performed CFA to confirm the model structure of original ABCD, the results showed that the model fit indices were mediocre: χ^2^ = 1019.254, *df* = 132, χ^2^/*df* = 7.722, *p* < 0.001; CFI = 0.736, IFI = 0.796, TLI = 0.762; RMSEA = 0.146 (90% CI 0.137 to 0.154).

The score of AVE for three subscales were higher than 0.5, in addition, all square root of AVE scores exceeded each of their correlations with other subscales which indicating appropriate convergent and discriminant validity (Table 3).

**Table 3:** Convergent and discriminant validity analysis.

ABCD-C	AVE	Inter-dimension correlations		
		RP	PA	DH
RP	0.671	**0.819**		
PA	0.603	0.140*	**0.777**	
DH	0.573	0.121*	0.368**	**0.757**

AVE, average variance extracted; ABCD-C, Chinese version of Attitudes and Beliefs about Cardiovascular Disease (ABCD) Risk Perception Questionnaire; RP, risk perception; PA, perceived benefits and intention to change physical activity; DH, perceived benefits and intention to change dietary habits.

Bold characters show the square of root of average variance extracted values.

*p < 0.05, **p < 0.001.

The significant positive correlation was founded between ABCD-C and one-item subjective CVD risk perception (*r* = 0.258, *P* < 0.001). The correlation coefficient of four dimensions (knowledge, risk perception, PA and Dietary) and subjective CVD risk perception was 0.043 (*P* = 0.446), 0.466 (*P* < 0.001), − 0.145 (*P* = 0.010), and 0.008 (*P* = 0.890), respectively.

#### Internal consistency and reliability

We calculated internal consistency estimates of reliability based on the factorial solution of EFA. The Cronbach’s *α* values of the four dimensions ranged from 0.801 to 0.940, the McDonald’s ω ranged from 0.853 to 0.952, which indicated acceptable and satisfactory internal consistency (Table S7). The corrected item-total subscale correlations ranged from 0.448 to 0.853, and no individual item was found to greatly increase the Cronbach’s α and McDonald’s ω if deleted (Table S8). For the 2-week test–retest reliability, 20 participants finished the second test. The test–retest reliability showed acceptable results (ICC = 0.788, 95% *CI* 0.527 to 0.902).

#### Item difficulty for Knowledge subscale of ABCD-C

The item difficulty index was 0.726 (ranging from 0.44 to 0.85), which means that the CVD Knowledge subscale has a moderate difficulty level (Table 4). Four items (2–5) showed an item difficulty index of more than 0.80, which means these items were easier for participants. Item 7 had the highest difficulty level (0.44); thus, more than half of the participants could not answer this question correctly. Regarding discrimination ability, the point-biserial coefficients of the items ranged from 0.572 to 0.707 (Table 4), which indicating sufficient discrimination ability.

**Table 4:** Item Statistic of Knowledge subscale of ABCD-C (*N* = 318, *M* ± *SD*).

No	Knowledge subscale Items	Difficulty Index	Discrimination power
1	Stress is one of the main causes of heart attack and stroke	0.701	0.659
2	Walking and gardening are considered types of exercise that can lower the risk of having a heart attack or stroke	0.852	0.572
3	Moderately intensive activity of 2.5 h a week will reduce your chance of having a heart attack or stroke	0.821	0.635
4	People who have diabetics are at a higher risk of having a heart disease or stroke	0.824	0.588
5	Managing your stress levels will help to manage your blood pressure	0.811	0.661
6	Heavy drinking can increase your cholesterol and triglyceride levels	0.780	0.656
7	High-density lipoprotein is the "good" cholesterol, and low-density lipoprotein is the "bad" cholesterol	0.440	0.707
8	A family history of heart disease is not a risk factor for high blood pressure	0.594	0.693

## Discussion

With the continuing burden of CVD worldwide, targeting the knowledge and risk perception of populations is critical to the performance of CVD primary and secondary prevention. Thus, a reliable and valid tool to measure CVD-related knowledge and subjective risk perception is needed. In response, Woringer et al*.* developed the ABCD Risk Perception Questionnaire. To the best of our knowledge, the ABCD Risk Perception Questionnaire had not been translated and validated in China. Thus, we conducted a cross-sectional study to cross-culturally adapt and validate the ABCD Risk Perception Questionnaire into Chinese. We found that the Chinese version of the ABCD Risk Perception Questionnaire was a reliable measurement. Thus, this questionnaire could be a valuable tool for public health providers, clinical practitioners, and researchers to evaluate an individual’s CVD knowledge and risk perception, which can be used to guide target CVD risk-reduction interventions.

The translation and adaptation processes were undertaken, following established guidelines^26^. When compared with the original scale, only two phrases were revised. In China, “five servings” as the diet unit is difficult to understand, as the definition of a “serving” varies. Hence, we revised “five servings” to “500 g,” according to the healthy diet recommendation of the Chinese CVD prevention guidelines^39^. Likewise, 2–1/2 h was revised to 2.5 h. Moreover, the total scale proved understandable and took about 6 to 10 min to complete.

We confirmed a three-factor structure for the 18-item scale, which accounted for 69.632% of the total variance; nevertheless, the pattern of factor loadings in our analysis was slightly different from the original one. The original subscales include perceived risk, perceived benefits and intention to change, and healthy eating intentions. The Hungarian version of the ABCD Risk Perception Questionnaire contained the same constructs as the original one, but the pattern of factor loadings varied. In addition, they created a simplified 10-item short version based on the factor loadings of CFA and item meaning, of which risk perception, perceived benefits and healthy eating habits containing 4, 3, 3 items respectively^20^. The recent Dutch version is a revision of the original ABCD Risk Perception Questionnaire, with two items added, and the subscale name was revised to Risk Perception, Perceived Benefits and Intention to Change Physical Activity, and Perceived Benefits and Intention to Change Dietary Habits, according to the factor analysis results^21^. In our analysis, Item 14 (“When I eat at least 500 g fruit and vegetables a day, I do something good for the health of my heart”), which was in Factor 2, showed a better loading to Factor 3. Further, we performed CFA to test the factorial structure, and the model fit indices were improved, which means that the item is logically related to Factor 3. This result was similar to the Dutch version^21^. In addition, we tested the model structure of the original ABCD questionnaire using our data, and the model fit indices were inferior to our model. Thus, we also revised the factor name in consideration of the factorial loading pattern, item meaning, and the Dutch version factor name, that is, Risk Perception, Perceived Benefits and Intention to Change Physical Activity, and Perceived Benefits and Intention to Change Dietary Habits.

The reliability evaluation showed that the ABCD -C was an acceptable instrument in this population. The Cronbach’s *α* value of ABCD-C was similar to those of the Dutch version (0.75, 0.93, 0.88, 0.84 for each of four dimensions)^21^ and slightly higher than those of the original scale (0.85, 0.82, 0.56 for last three dimensions)^19^ and Hungarian version (0.504, 0.945, 0.822, 0.756 for each of four dimensions)^20^. Cronbach’s *α* coefficients are an inherent property of the response pattern of a specific sample and not a characteristic of the scale itself^30^. Therefore, it is important to measure Cronbach’s *α* when validating an instrument in a different sample from that of the original participants. The results also indicated that the ABCD Risk Perception Questionnaire was a reliable instrument when applied to Chinese population. In addition, we reported a higher Cronbach’s *α* value of risk perception dimension beyond *Tavakol *et al*.* recommendation^30^, it may suggest that some items were redundant as they were testing the same question but in a different guise. Further study would conduct to shorten item according to semantic and subjective judgment of expert team, and explore the internal consistency between shorter version and original versions.

The item difficulty analysis showed that the Knowledge subscale had a moderate difficulty level and a potential discriminatory capability. The CVD Knowledge subscale contains four easy items, three moderate items, and one difficult item; thus, it can differentiate participants sufficiently and provide an understanding of the individual’s CVD knowledge level. The knowledge score (46.8% participants had an accuracy rate higher than 80%) was slightly higher than that of other studies (28.85%)^40^, possibly due to that the education level of participants in our study were higher than in previous study.

The current study has several limitations. First, the study participants were recruited from a city in south China by convenience sampling and, this may lead to selection bias. Thus, the generalizability of the results of this study might be threatened, as a large number of the participants were recruited from Han ethic group and higher level of education. Further research should test the ABCD-C in more diverse sample from various regions of China. Second, we did not conduct CFA on a separate sample to confirm the structure of the factors resulting from the EFA. The results of CFA in our study will favor the EFA model and inflate the model fit indices. As such, we will test this factor structure in further study. Third, the concurrent validity was performed based on correlations between the scores of ABCD-C and single item CVD subjective risk perception which was widely used to evaluate CVD risk perception in previous studies^16,41^. It will be beneficial to analyze the relationship between ABCD-C and objective estimated CVD risk (e.g. prediction for atherosclerotic cardiovascular disease risk in China, China-PAR^42^) in further research, which allows conclusion of underestimation, overestimation or accurate risk perception to be drawn.

## Conclusion

This is the first Chinese version of the ABCD Risk Perception Questionnaire. The study followed a robust methodology in its translation and validation. This study confirmed that the ABCD-C has good psychometric properties to measure CVD-related knowledge and risk perception in the Chinese adult population.

## Supplementary Information

Supplementary Information.

## Data Availability

The data will be shared upon reasonable request to the corresponding author.
