# Supplementary material for: Psychometric properties of the Chinese version of Attitudes and Beliefs about Cardiovascular Disease Risk Perception Questionnaire
Source: Sci Rep. 2022 Nov 24;12:20241. doi: 10.1038/s41598-022-24620-9 (PMC9691742; doi:10.1038/s41598-022-24620-9)
Supplement: Supplementary file 1 — Supplementary Information. [file 41598_2022_24620_MOESM1_ESM.docx]

**Supplementary materials**

**Contents**

**Characteristics of respondents**

**Item frequencies**

- **Knowledge subscale**
- **Risk subscale**

**Inter-item correlations**

**Correlations between four dimensions**

**Reliability analysis**

**Characteristics of respondents**

A total of 341 participants were approached for the study. Of these, 93 (27.3%) completed the paper-based questionnaire, and seven answers (2.0%) had more than three blanks and were excluded. A total of 248 participants (72.7%) finished the online survey, for which five responses had a completion time of less than 2 minutes, and 11 responses had very similar option choices for most items; thus, the data from 16 questionnaires (4.7%) were excluded.

No difference in valid response rate was found between paper-based and online participants (*χ^2^*=0.102, *P*=0.750). Other characteristics of responders between paper-based survey and online survey were showed in table S1.

Table S1 Characteristics of responders between paper and online survey (*n* = 318)

| Characteristic | *N* (%) | Paper-based survey | Online survey | *χ^2^*/ANOVA F (*p* value) |
| --- | --- | --- | --- | --- |
| Total sample | 318 | 87(27.4) | 231(72.6) |  |
| Age |  | 56.65±17.73 | 36.62±12.93 | 121.687(<0.001) ^a^ |
| Sex |  |  |  |  |
| Male | 134 (42.1) | 42(13.2) | 92(28.9) | 2.170 (0.141) |
| Female | 184 (57.9) | 44(13.8) | 140(44.1) |  |
| Education level |  |  |  | 43.709(<0.001) |
| Junior school or below | 44 (13.8) | 29(9.1) | 15(4.7) |  |
| High school/specialized secondary school | 59 (18.6) | 17(5.3) | 42(13.3) |  |
| Specialty/bachelor | 193 (60.7) | 33(10.4) | 160(50.3) |  |
| Postgraduate or above | 22 (6.9) | 7(2.2) | 15(4.7) |  |
| Ethnic group |  |  |  | 0.186(0.667) |
| Han Chinese | 309 (97.2) | 83(26.1) | 226(71.1) |  |
| Minorities | 9 (2.8) | 3(0.9) | 6(1.9) |  |
| Employment status |  |  |  | 27.247(<0.001) |
| Employed | 193 (60.7) | 32(10.1) | 161(50.6) |  |
| Unemployed | 125 (39.3) | 54(17.0) | 71(22.3) |  |
| Smoking status |  |  |  | 1.549(0.213) |
| Current smoking | 43 (13.5) | 15(4.7) | 28(8.8) |  |
| Non-smoking/quit smoking | 275 (86.5) | 71(22.3) | 204(64.2) |  |
| Drinking status |  |  |  | 0.391(0.532) |
| Current drinking | 55 (17.3) | 13(4.1) | 42(13.2) |  |
| Non-drinking/quit drinking | 263 (82.7) | 73(23.0) | 190(59.7) |  |
| CVD family history |  |  |  | 1.313(0.252) |
| Yes | 28 (8.8) | 5(1.6) | 23(7.2) |  |
| No | 290 (91.2) | 81(25.5) | 209(65.7) |  |
| Subjective health status |  |  |  | 9.528(0.023) |
| Excellent | 120 (37.7) | 22(6.9) | 98(30.8) |  |
| Good | 154 (48.4) | 46(14.4) | 108(34.0) |  |
| Fair | 39 (12.3) | 16(5.1) | 23(7.2) |  |
| Poor | 5 (1.6) | 2(0.6) | 3(1.0) |  |
| ABCD-C |  | 54.91±6.32 | 53.65±8.75 | 5.437(0.020) ^a^ |
| knowledge |  | 5.57±2.28 | 5.92±2.16 | 3.649(0.057) ^a^ |
| RP |  | 18.09±4.00 | 17.01±5.52 | 4.493(0.035) ^a^ |
| PA |  | 18.36±4.08 | 18.02±4.20 | 0.228(0.633) ^a^ |
| DH |  | 12.90±2.04 | 12.71±2.27 | 0.020(0.889) ^a^ |

*Note.* ^a^ ANOVA analysis. RP, risk perception; PA, perceived benefits and intention to change physical activity; DH, perceived benefits and intention to change dietary habits; CVD, cardiovascular disease.

Twenty participants who were randomly selected from the total sample in a two-week interval to participate in our survey again to calculate the test-retest reliability. Table S2 showed the characteristics of these participants.

Table S2 Characteristics of participants for test-retest reliability (*n*=20)

| Characteristic | Mean±SD*/N* (%) |
| --- | --- |
| Age | 45.74±18.39 |
| Sex |  |
| Male | 5(25.0) |
| Female | 15 (75.0) |
| Education level |  |
| Junior school or below | 6 (30.0) |
| High school/specialized secondary school | 1 (5.0) |
| Specialty/bachelor | 10 (50.0) |
| Postgraduate or above | 3 (15.0) |
| Ethnic group |  |
| Han Chinese | 18 (90.0) |
| Minorities | 2 (10.0) |
| Employment status |  |
| Employed | 10 (50.0) |
| Unemployed | 10 (50.0) |
| Smoking status |  |
| Current smoking | 2 (10.0) |
| Non-smoking/quit smoking | 18 (90.0) |
| Drinking status |  |
| Current drinking | 3 (15.0) |
| Non-drinking/quit drinking | 17 (85.0) |
| CVD family history |  |
| Yes | 4 (20.0) |
| No | 16 (80.0) |
| Subjective health status |  |
| Excellent | 8 (40.0) |
| Good | 8 (40.0) |
| Fair | 3 (15.0) |
| Poor | 1 (5.0) |
| ABCD-C | 49.95±8.06 |
| knowledge | 5.70±1.92 |
| RP | 17.25±4.51 |
| PA | 15.20±3.93 |
| DH | 11.80±3.05 |

*Note.* RP, risk perception; PA, perceived benefits and intention to change physical activity; DH, perceived benefits and intention to change dietary habits; CVD, cardiovascular disease.

**Item frequencies**

**Knowledge subscale**

The tendency of correct answers in the individual knowledge questions are presented in table S3. The differences between items in response frequencies indicates the difference in the item difficulty level. Two hundred seventy-one participants (85.2%) know that walking and gardening are considered types of exercise that can lower the risk of having a heart attack or stroke. While only 44.0% participants know the clinical significance of high- and low-density lipoprotein.

Table S3 Item response frequencies for knowledge subscale (*n*=318)

| **No** | **Knowledge questions** | **Responded correctly** | |
| --- | --- | --- | --- |
|  |  | **Frequency** | **(%)** |
| 1 | Stress is one of the main causes of heart attack and stroke. | 223 | 70.1 |
| 2 | Walking and gardening are considered types of exercise that can lower the risk of having a heart attack or stroke. | 271 | 85.2 |
| 3 | Moderately intensive activity of 2.5 hours a week will reduce your chance of having a heart attack or stroke. | 261 | 82.1 |
| 4 | People who have diabetics are at a higher risk of having a heart disease or stroke. | 262 | 82.4 |
| 5 | Managing your stress levels will help to manage your blood pressure. | 258 | 81.1 |
| 6 | Heavy drinking can increase your cholesterol and triglyceride levels. | 248 | 78.0 |
| 7 | High-density lipoprotein is the "good" cholesterol, and low-density lipoprotein is the "bad" cholesterol. | 140 | 44.0 |
| 8 | A family history of heart disease is not a risk factor for high blood pressure. | 189 | 59.4 |

**Risk subscale**

The frequency and percentage of participants response options of the risk subscale are presented in table S4. Almost all items have moderate variation to differentiate respondents. Six items (item12-14, 16, 22, 24) shows less five responses in the ‘strongly agree’ or ‘strongly disagree’ option.

Table S4 Item response frequencies for the risk subscale [*n*=318, n(%)]

| No | Risk scale items | Strongly Disagree | Disagree | Agree | Strongly Agree | NA |
| --- | --- | --- | --- | --- | --- | --- |
| 9 | I feel I will suffer a heart disease/stroke someday in my life. | 25(7.9) | 109(34.3) | 160(50.3) | 14(4.4) | 10(3.1) |
| 10 | It`s likely that I will have a heart disease or stroke in the future. | 29(9.1) | 164(51.6) | 107(33.6) | 9(2.8) | 9(2.8) |
| 11 | It`s likely that I will have a heart disease or stroke at some time in my life. | 32(10.1) | 169(53.1) | 96(30.2) | 11(3.5) | 10(3.1) |
| 12 | It`s possible that I will have a heart disease or stroke within the next 10 years. | 46(14.5) | 200(62.9) | 60(18.9) | 3(0.9) | 9(2.8) |
| 13 | The chance that I will have a heart disease or stroke within the next 10 years is high. | 43(13.5) | 202(63.5) | 61(19.2) | 4(1.3) | 8(2.5) |
| 14 | I will probably have a heart attack or stroke due to my past and/or current lifestyle behavior. | 44(13.8) | 169(53.1) | 94(29.6) | 2(0.6) | 9(2.8) |
| 15 | I am not worried that I might have a heart attack or stroke. | 27(8.5) | 63(19.8) | 156(49.1) | 66(20.8) | 6(1.9) |
| 16 | I am concerned because there is a chance that I will have a heart attack or stroke in the near future. | 42(13.2) | 180(55.6) | 86(27.0) | 4(1.3) | 6(1.9) |
| 17 | I am considering exercising at least 2.5 hours a week. | 11(3.5) | 45(14.2) | 182(57.2) | 71(22.3) | 9(2.8) |
| 18 | I intend or want to exercise at least 2.5 hours a week. | 11(3.5) | 52(16.4) | 169(63.1) | 79(24.8) | 7(2.2) |
| 19 | When I exercise at least 2.5 hours a week, I do something good for the health of my heart. | 10(3.1) | 20(6.3) | 155(48.7) | 125(39.3) | 8(2.5) |
| 20 | I am confident that I can gain or maintain a healthy weight by exercising at least 2.5 hours a week in the next 2 months. | 14(4.4) | 66(20.80 | 163(51.3) | 68(21.4) | 7(2.2) |
| 21 | I am not considering exercising 2.5 hours a week. | 68(21.4) | 177(55.7) | 59(18.6) | 10(3.1) | 4(1.3) |
| 22 | When I eat at least 500g fruits and vegetables a day, I do something good for the health of my heart. | 4(1.3) | 9(2.8) | 205(64.5) | 99(31.1) | 1(0.3) |
| 23 | Increasing my exercise to at least 2.5 hours a week will reduce my chance of having a heart attack or stroke. | 10(3.1) | 23(7.2) | 146(45.9) | 136(42.8) | 3(0.9) |
| 24 | I am confident that I can eat at least 500g fruits and vegetables per day in the next 2 months. | 4(1.3) | 45(14.2) | 169(53.1) | 97(30.5) | 3(0.9) |
| 25 | I am considering eating at least 500g fruits and vegetables a day. | 7(2.2) | 30(9.4) | 192(60.4) | 88(27.7) | 1(0.3) |
| 26 | I am not considering eating at least 500g fruits and vegetables a day. | 121(38.1) | 169(53.1) | 22(6.9) | 6(1.9) | 0(0.0) |

**Inter-item correlations**

The correlation matrix indicate that strong correlation exists between item 7 and 8 (r=0.733). Moderate correlation was observed between item 2 and 3 (r=0.591), item 3 and 4 (r=0.580), item 2 and 4 (r=0.529). The correlation matrix indicate that strong correlation exists between item 9 and 10 (r=0.810), item 10 and 11 (r=0.862), item 7 and 8 (r=0.847). Moderate correlation was observed between item 9 and 11 (r=0.754), item 10 and 14 (r=0.754), item 12 and 13 (r=0.778), item 11 and 14 (0.706), et al.

The negative correlation was observed between several knowledge items and risk perception subscale items (Table S5).

Table S5 Inter-item correlations of ABCD-C

|  | Item1 | Item2 | Item3 | Item4 | Item5 | Item6 | Item7 | Item8 | Item9 | Item10 | Item11 | Item12 | Item13 | Item14 | Item15 | Item16 | Item17 | Item18 | Item19 | Item20 | Item21 | Item22 | Item23 | Item24 | Item25 | Item26 |
| --- | --- | --- | --- | --- | --- | --- | --- | --- | --- | --- | --- | --- | --- | --- | --- | --- | --- | --- | --- | --- | --- | --- | --- | --- | --- | --- |
| Item1 | 1.000 |  |  |  |  |  |  |  |  |  |  |  |  |  |  |  |  |  |  |  |  |  |  |  |  |  |
| Item2 | 0.232 | 1.000 |  |  |  |  |  |  |  |  |  |  |  |  |  |  |  |  |  |  |  |  |  |  |  |  |
| Item3 | 0.25 | 0.591 | 1.000 |  |  |  |  |  |  |  |  |  |  |  |  |  |  |  |  |  |  |  |  |  |  |  |
| Item4 | 0.239 | 0.529 | 0.58 | 1.000 |  |  |  |  |  |  |  |  |  |  |  |  |  |  |  |  |  |  |  |  |  |  |
| Item5 | 0.511 | 0.207 | 0.278 | 0.241 | 1.000 |  |  |  |  |  |  |  |  |  |  |  |  |  |  |  |  |  |  |  |  |  |
| Item6 | 0.516 | 0.249 | 0.227 | 0.253 | 0.423 | 1.000 |  |  |  |  |  |  |  |  |  |  |  |  |  |  |  |  |  |  |  |  |
| Item7 | 0.302 | 0.244 | 0.299 | 0.21 | 0.347 | 0.334 | 1.000 |  |  |  |  |  |  |  |  |  |  |  |  |  |  |  |  |  |  |  |
| Item8 | 0.314 | 0.125 | 0.232 | 0.173 | 0.420 | 0.380 | 0.733 | 1.000 |  |  |  |  |  |  |  |  |  |  |  |  |  |  |  |  |  |  |
| Item9 | -0.235 | -0.063 | -0.024 | -0.118 | -0.146 | -0.124 | -0.03 | 0.039 | 1.000 |  |  |  |  |  |  |  |  |  |  |  |  |  |  |  |  |  |
| Item10 | -0.113 | -0.074 | -0.043 | -0.056 | -0.086 | -0.077 | -0.011 | 0.072 | 0.810 | 1.000 |  |  |  |  |  |  |  |  |  |  |  |  |  |  |  |  |
| Item11 | -0.046 | -0.003 | 0.029 | -0.014 | -0.036 | -0.053 | 0.016 | 0.071 | 0.754 | 0.862 | 1.000 |  |  |  |  |  |  |  |  |  |  |  |  |  |  |  |
| Item12 | -0.162 | -0.034 | 0.004 | -0.008 | -0.123 | -0.115 | 0.010 | 0.072 | 0.652 | 0.689 | 0.656 | 1.000 |  |  |  |  |  |  |  |  |  |  |  |  |  |  |
| Item13 | -0.139 | -0.084 | -0.038 | -0.063 | -0.118 | -0.141 | 0.024 | 0.075 | 0.659 | 0.653 | 0.582 | 0.778 | 1.000 |  |  |  |  |  |  |  |  |  |  |  |  |  |
| Item14 | -0.177 | -0.055 | -0.017 | -0.04 | -0.11 | -0.123 | -0.066 | 0.031 | 0.692 | 0.754 | 0.706 | 0.681 | 0.724 | 1.000 |  |  |  |  |  |  |  |  |  |  |  |  |
| Item15 | -0.179 | -0.17 | -0.137 | -0.121 | -0.176 | -0.113 | -0.029 | 0.084 | 0.515 | 0.575 | 0.492 | 0.527 | 0.557 | 0.682 | 1.000 |  |  |  |  |  |  |  |  |  |  |  |
| Item16 | -0.154 | -0.137 | -0.113 | -0.08 | -0.162 | -0.129 | 0.012 | 0.100 | 0.596 | 0.651 | 0.577 | 0.655 | 0.660 | 0.772 | 0.847 | 1.000 |  |  |  |  |  |  |  |  |  |  |
| Item17 | -0.134 | -0.025 | 0.008 | -0.077 | -0.087 | -0.036 | -0.087 | -0.047 | 0.283 | 0.182 | 0.183 | 0.121 | 0.235 | 0.197 | 0.015 | 0.055 | 1.000 |  |  |  |  |  |  |  |  |  |
| Item18 | -0.054 | 0.068 | 0.001 | -0.017 | 0.009 | 0.092 | 0.008 | 0.004 | 0.186 | 0.179 | 0.144 | 0.079 | 0.161 | 0.087 | -0.012 | 0.015 | 0.763 | 1.000 |  |  |  |  |  |  |  |  |
| Item19 | -0.139 | 0.020 | -0.001 | -0.021 | -0.087 | -0.117 | -0.093 | -0.06 | 0.228 | 0.098 | 0.101 | -0.028 | 0.083 | 0.067 | -0.018 | -0.033 | 0.629 | 0.585 | 1.000 |  |  |  |  |  |  |  |
| Item20 | -0.110 | 0.051 | -0.004 | -0.068 | -0.017 | 0.032 | 0.027 | 0.021 | 0.226 | 0.146 | 0.166 | 0.094 | 0.147 | 0.126 | -0.021 | -0.015 | 0.699 | 0.648 | 0.550 | 1.000 |  |  |  |  |  |  |
| Item21 | -0.008 | 0.040 | -0.022 | -0.042 | 0.057 | 0.114 | 0.001 | -0.003 | 0.093 | 0.147 | 0.099 | 0.075 | 0.107 | 0.056 | 0.034 | 0.010 | 0.594 | 0.722 | 0.501 | 0.555 | 1.000 |  |  |  |  |  |
| Item22 | -0.039 | 0.129 | 0.112 | 0.096 | 0.066 | 0.056 | 0.034 | 0.043 | 0.143 | 0.038 | 0.071 | 0.027 | 0.034 | 0.050 | -0.033 | -0.044 | 0.259 | 0.274 | 0.335 | 0.279 | 0.111 | 1.000 |  |  |  |  |
| Item23 | -0.102 | 0.071 | 0.011 | 0.070 | -0.092 | -0.081 | -0.103 | -0.095 | 0.191 | 0.089 | 0.122 | 0.042 | 0.121 | 0.060 | -0.080 | -0.036 | 0.530 | 0.506 | 0.706 | 0.524 | 0.489 | 0.264 | 1.000 |  |  |  |
| Item24 | -0.114 | 0.108 | 0.09 | 0.100 | -0.047 | -0.073 | -0.003 | 0.002 | 0.274 | 0.224 | 0.262 | 0.208 | 0.217 | 0.217 | 0.050 | 0.108 | 0.247 | 0.259 | 0.262 | 0.356 | 0.170 | 0.546 | 0.347 | 1.000 |  |  |
| Item25 | -0.068 | 0.104 | 0.136 | 0.111 | -0.003 | -0.066 | -0.028 | -0.031 | 0.153 | 0.071 | 0.102 | 0.084 | 0.111 | 0.063 | 0.010 | 0.031 | 0.253 | 0.287 | 0.361 | 0.256 | 0.155 | 0.674 | 0.362 | 0.683 | 1.000 |  |
| Item26 | 0.113 | 0.117 | 0.178 | 0.139 | 0.161 | 0.137 | 0.073 | 0.117 | 0.022 | 0.028 | 0.101 | -0.004 | -0.005 | 0.007 | -0.024 | -0.065 | 0.164 | 0.197 | 0.108 | 0.228 | 0.137 | 0.348 | 0.182 | 0.493 | 0.559 | 1.000 |

**Correlations between four dimensions**

Of 26 items of ABCD-C, 8 items of knowledge dimensions with ‘true’ or ‘false/do not know’ scoring could not be entered into factor analysis. Thus, the convergent and discriminant validity analysis did not include knowledge dimension. Then, we conducted further correlation analysis between four factors. The results demonstrated negative correlations with CVD knowledge dimension and RP, PA factors; positive correlation was observed between CVD knowledge subscale and DH factor (table S6).

Table S6 Correlations between four dimensions of ABCD-C

| ABCD-C |  | Knowledge | RP | PA | DH |
| --- | --- | --- | --- | --- | --- |
| Knowledge | Correlation Coefficient | 1.000 |  |  |  |
|  | Sig. (2-tailed) | / |  |  |  |
| RP | Correlation Coefficient | -0.110 | 1.000 |  |  |
|  | Sig. (2-tailed) | 0.050 | / |  |  |
| PA | Correlation Coefficient | -0.050 | 0.140* | 1.000 |  |
|  | Sig. (2-tailed) | 0.379 | 0.013 | / |  |
| DH | Correlation Coefficient | 0.090 | 0.121* | 0.368** | 1.000 |
|  | Sig. (2-tailed) | 0.107 | 0.030 | <0.001 | / |

*Note.* ABCD-C, Chinese version of Attitudes and Beliefs about Cardiovascular Disease (ABCD) Risk Perception Questionnaire; RP, risk perception; PA, perceived benefits and intention to change physical activity; DH, perceived benefits and intention to change dietary habits.

Pearson correlation was performed. **p* < 0.05, ***p* < 0.001.

**Reliability analysis**

The Cronbach’s α coefficient of the original ABCD questionnaire and ABCD-C were showed in table S7. The McDonald’s ω coefficient and ICC value of ABCD-C was also showed in table S7.

Table S7 Reliability analysis

| ABCD-C | Dimension1 | Dimension2 | Dimension3 | Dimension4 |
| --- | --- | --- | --- | --- |
| Cronbach’s α coefficient | 0.801 | 0.940 | 0.900 | 0.830 |
| McDonald’s ω coefficient | 0.853 | 0.952 | 0.923 | 0.888 |
| Intraclass correlation coefficient | 0.852 | 0.830 | 0.781 | 0.698 |
| Original ABCD | Dimension1 | Dimension2 | Dimension3 | Dimension4 |
| Cronbach’s α coefficient | / | 0.85 | 0.82 | 0.56 |

*Note.* Dimension1, knowledge; Dimension2, risk perception; Dimension3, perceived benefits and intention to change physical activity; Dimension4, perceived benefits and intention to change dietary habits; ABCD, the original Attitudes and Beliefs about Cardiovascular Disease (ABCD) Risk Perception Questionnaire by *Woringer et al*; ABCD-C, Chinese version of ABCD questionnaire.

The corrected item-total subscale correlations ranged from 0.448 to 0.853, and no individual item was found to greatly increase the Cronbach’s α and McDonald’s ω if deleted, so all items were retained (Table S8).

Table S8 item analysis

| Dimension | Item | Item-total correlation | Cronbach’s α if item is deleted | McDonald’s ω if item is deleted |
| --- | --- | --- | --- | --- |
| Knowledge | 1 | 0.513 | 0.779 | 0.842 |
|  | 2 | 0.448 | 0.788 | 0.846 |
|  | 3 | 0.512 | 0.779 | 0.84 |
|  | 4 | 0.455 | 0.787 | 0.845 |
|  | 5 | 0.541 | 0.775 | 0.84 |
|  | 6 | 0.527 | 0.776 | 0.841 |
|  | 7 | 0.562 | 0.771 | 0.839 |
|  | 8 | 0.545 | 0.774 | 0.841 |
| Risk perception | 9 | 0.788 | 0.932 | 0.947 |
|  | 10 | 0.852 | 0.927 | 0.944 |
|  | 11 | 0.780 | 0.932 | 0.947 |
|  | 12 | 0.777 | 0.933 | 0.947 |
|  | 13 | 0.772 | 0.933 | 0.948 |
|  | 14 | 0.853 | 0.927 | 0.944 |
|  | 15 | 0.694 | 0.940 | 0.952 |
|  | 16 | 0.810 | 0.930 | 0.946 |
| perceived benefits and intention to change physical activity | 17 | 0.792 | 0.873 | 0.908 |
|  | 18 | 0.792 | 0.873 | 0.908 |
|  | 19 | 0.717 | 0.885 | 0.916 |
|  | 20 | 0.722 | 0.884 | 0.915 |
|  | 21 | 0.689 | 0.889 | 0.918 |
|  | 23 | 0.660 | 0.893 | 0.921 |
| perceived benefits and intention to change dietary habits | 22 | 0.619 | 0.804 | 0.885 |
|  | 24 | 0.695 | 0.769 | 0.868 |
|  | 25 | 0.795 | 0.720 | 0.844 |
|  | 26 | 0.543 | 0.835 | 0.903 |
